# *C9ORF72* patient-derived endothelial cells drive blood-brain barrier disruption and contribute to neurotoxicity

**DOI:** 10.1186/s12987-024-00528-6

**Published:** 2024-04-11

**Authors:** Ana Aragón-González, Allan C Shaw, Jannigje R Kok, Florence S Roussel, Cleide dos Santos Souza, Sarah M Granger, Tatyana Vetter, Yolanda de Diego, Kathrin C Meyer, Selina N Beal, Pamela J Shaw, Laura Ferraiuolo

**Affiliations:** 1 Sheffield Institute for Translational Neuroscience, University of Sheffield, 385 Glossop Road, S10 2HQ Sheffield, UK; 2 Facultad de Medicina, Universidad de Málaga, 29010 Malaga, Spain; 3 Center for Gene Therapy, The Abigail Wexner Research Institute, Nationwide Children’s Hospital, OH 43205 Columbus, USA; 4 Department of Pediatrics, The Ohio State University, Columbus, OH USA; 5 Research Group PAIDI CTS-546, Institute of Biomedical Research of Málaga (IBIMA), 29010 Malaga, Spain; 6 Department of Cell Biology, Physiology, and Immunology, University of Córdoba, Campus Rabanales, Cordoba, Spain; 7 NIHR Sheffield Biomedical Research Centre, Sheffield Teaching Hospitals NHS Foundation Trust, Glossop Road, Sheffield, UK

**Keywords:** Stem cells, Blood brain barrier, Amyotrophic lateral sclerosis, C9ORF72, *In vitro* modelling, Neurodegeneration

## Abstract

The blood-brain barrier (BBB) serves as a highly intricate and dynamic interface connecting the brain and the bloodstream, playing a vital role in maintaining brain homeostasis. BBB dysfunction has been associated with multiple neurodegenerative diseases, including amyotrophic lateral sclerosis (ALS); however, the role of the BBB in neurodegeneration is understudied. We developed an ALS patient-derived model of the BBB by using cells derived from 5 patient donors carrying *C9ORF72* mutations. Brain microvascular endothelial-like cells (BMEC-like cells) derived from *C9ORF72*-ALS patients showed altered gene expression, compromised barrier integrity, and increased P-glycoprotein transporter activity. In addition, mitochondrial metabolic tests demonstrated that *C9ORF72*-ALS BMECs display a significant decrease in basal glycolysis accompanied by increased basal and ATP-linked respiration. Moreover, our study reveals that C9-ALS derived astrocytes can further affect BMECs function and affect the expression of the glucose transporter Glut-1. Finally, *C9ORF72* patient-derived BMECs form leaky barriers through a cell-autonomous mechanism and have neurotoxic properties towards motor neurons.

**Graphical Abstract:**

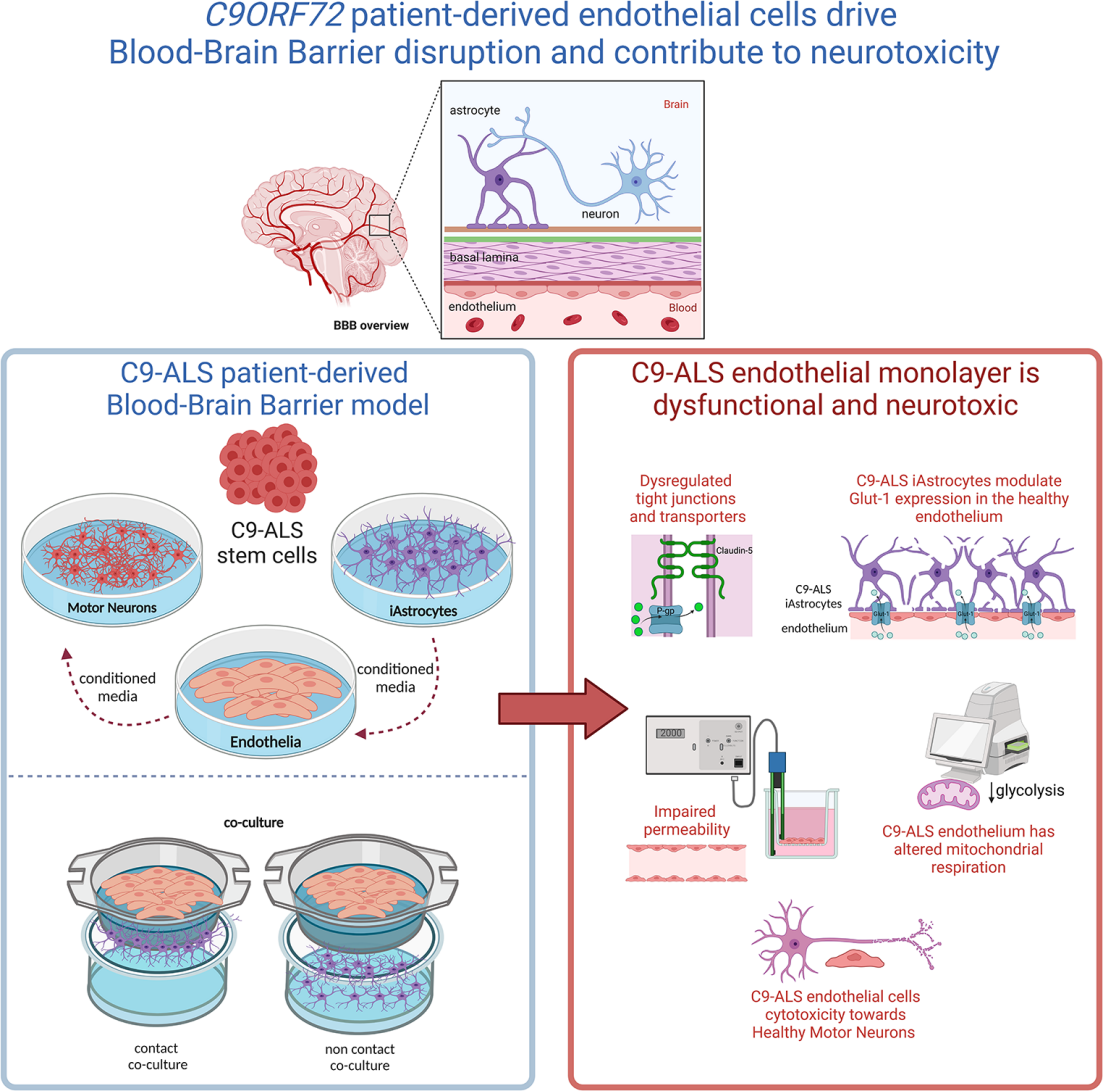

**Supplementary Information:**

The online version contains supplementary material available at 10.1186/s12987-024-00528-6.

## Introduction

The blood-brain barrier (BBB) serves as a highly intricate and dynamic interface connecting the brain and the bloodstream, carrying out several specialised functions. It comprises endothelial cells lining the capillary wall, astrocyte end-feet enveloping the capillary, and pericytes embedded in the capillary basement membrane [1]. The BBB permits the passive passage of hydrophobic molecules such as O_2_, CO_2_, and hormones, as well as small polar molecules. It regulates the exchange of metabolic substrates, such as glucose, using specific transport proteins across the barrier. Whilst tight regulation of molecular exchange between the blood and the brain is crucial for central nervous system function, the endothelial interface limits the entry of neuro-therapeutic molecules, thus making manipulations of BBB permeability an interesting target [2]. Conversely, disruptions in the BBB commonly occur in neurodegenerative diseases like Alzheimer’s (AD) and amyotrophic lateral sclerosis (ALS). Despite growing evidence of BBB dysfunction in these conditions, further research is needed to understand its role in disease progression [3].

Brain microvascular endothelial cells (BMECs) are the major cellular element in the BBB [4]. These cells present unique characteristics that distinguish them from the vascular endothelium in the rest of the body. BMEC-like cells lack fenestrations, express tight junctions (TJs) and specialised transporters, and interact with other cell types comprising the neurovascular unit (NVU). These attributes allow them to regulate the movement of ions, molecules, and cells between the blood and the brain [5].

The other main component of the BBB is the astrocyte [6], which plays an essential role in establishing and maintaining it. Astrocytic end-feet form a coating network around the brain vasculature, the glia limitans, and, together with endothelial cells and pericytes [7], they form the BBB, separating the bloodstream from the brain parenchyma. All components interact with each other, contributing to BBB function, development, and maintenance [4]. It is well documented that astrocyte function dysregulation is associated with neurodegenerative diseases such as ALS [8], AD [9]; and paediatric neurological disorders like Rett syndrome [10]. ALS, also known as motor neuron disease (MND), is a fatal neurodegenerative disorder characterised by progressive loss of upper and lower motor neurons (MNs), causing progressive paralysis, muscle atrophy due to denervation and, consequently, death due to respiratory failure. To date, there is no effective drug treatment for ALS. At present, Riluzole, Edaravone and Relyvrio are the only drugs approved by the US Food and Drugs Administration (FDA), providing modest benefits only in some patients [11].

Although most cases of ALS are sporadic (sALS) in origin, meaning that there is no family history of the disease, about 10% of patients are familial (fALS). Even though more than 35 genes have been linked with fALS, the most common disease-causing mutations are hexanucleotide (GGGGCC) intronic repeat expansions in the *C9ORF72* (C9) gene, followed by mutations in *SOD1* [12]. Remarkably, familial and sporadic ALS are for the most part clinically indistinguishable. Importantly, *C9ORF72* mutations are also known to cause frontotemporal dementia (FTD). Although the link between ALS pathology and BBB dysfunction is not clear, transgenic rodents expressing human *SOD1* mutations develop a leaky BBB with higher permeability, enlarged astrocytic end-feet, and an interrupted basement membrane associated with a reduction of BMEC-like cells and astrocytes, thus leading to oedema and microbleeds. This pathological phenotype is also observed in ALS patients [13]. The early BBB breakdown might appear as cerebral microbleeds, which are frequently seen in vascular dementia [14] and AD patients [15], suggesting that BBB failure might precede neurodegeneration.

Although vascular pathology has not been frequently studied in C9 mutation carriers, a recent publication reported increased glucose transport, together with enhanced Glucose-1 transporter expression in the BBB of a C9-ALS mouse model. In contrast, other permeability processes, such as passive diffusion or efflux transport, remained unaffected [16]. Consistently, P-glycoprotein transport was not altered in C9-ALS mice, and only a mild increase in ZO-1 expression was reported [16]. In addition, Sweeney et al. 2019 [17] proposed that BBB disruption due to endothelial cell degeneration causes extravasation of erythrocytes and accumulation of plasma-derived proteins such as Immunoglobulin G (IgG).

Currently, there is limited information available on how BBB breakdown affects the progression of ALS. However, a cross-sectional study by Prell et al. [18], focussing on cerebrospinal fluid and blood samples taken from ALS patients, found that there is no correlation between higher disease aggressiveness and markers of BBB breakdown. Thus, indicating that this pathological phenotype might be secondary to neurodegeneration.

Considering the knowledge gap in this area of ALS/FTD pathology and the complexity of dissecting the contribution of different cell types to the neurodegenerative process, the present study sought to interrogate a human *C9ORF72* patient-derived model of the BBB to test the leakiness of such a system and unravel the role of endothelial cells in BBB dysregulation. We demonstrated that *C9ORF72* patient-derived endothelial cells form leaky barriers through a cell-autonomous mechanism and have neurotoxic properties towards healthy motor neurons.

## Results

### Hi-PSC-derived BMEC-like cells express a vascular/endothelial profile and show barrier functionality

**Fig. 1:**
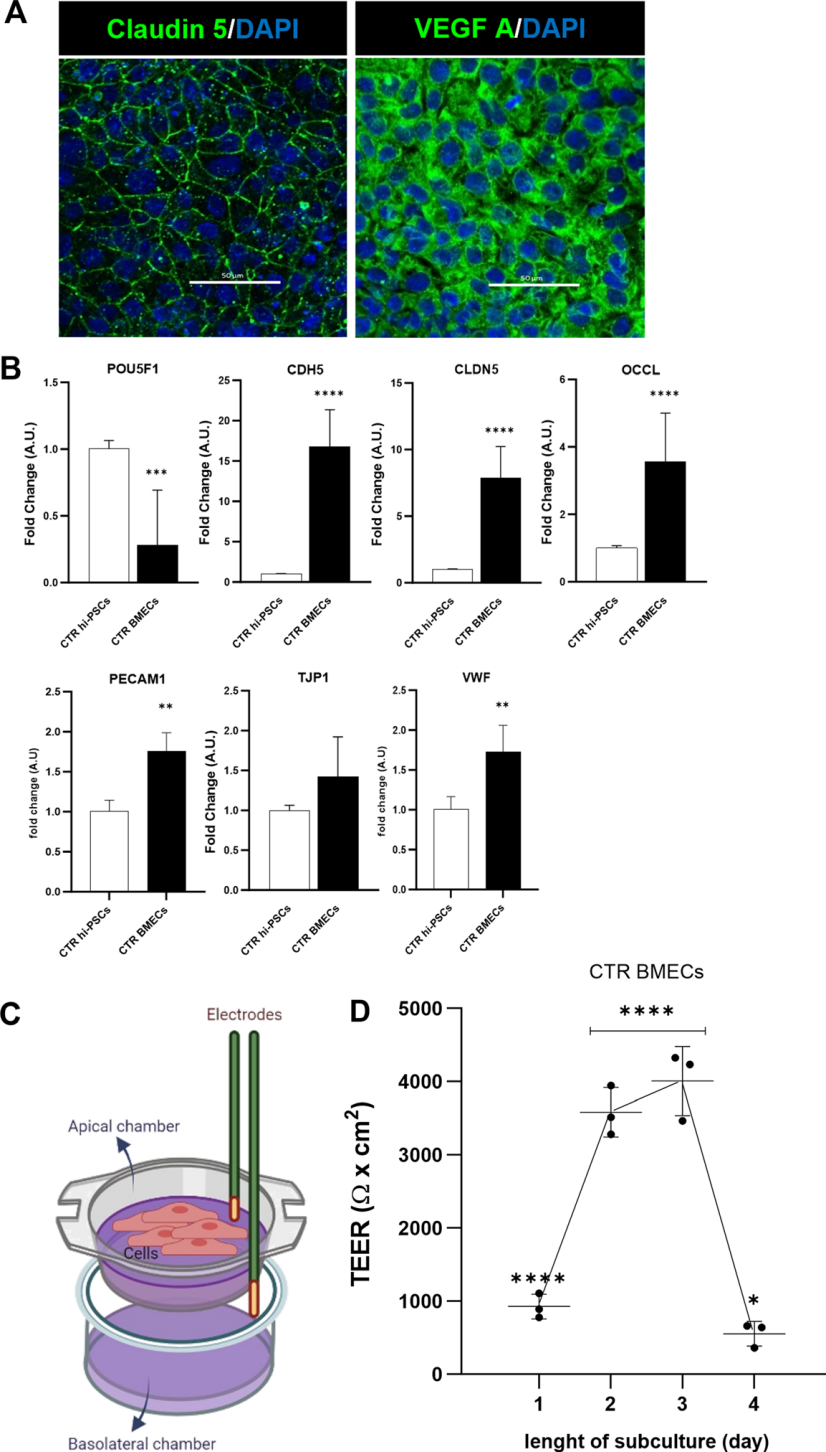
BMEC-like cells can be differentiated from hi-PSCs and can produce a cell barrier *in vitro.* **A**) Immunocytochemistry of BMEC cells differentiated from a healthy hi-PSCs donor. Claudin-5 and Vascular endothelial growth factor A (VEGF-A) using the RA-enhanced Lippmann’s laboratory protocol from 2019 [19]. Images were acquired with a Nikon confocal microscope. Scale bar 50 μm. **B**) Transcriptional expression of pluripotency and endothelial-specific markers in hi-PSCs and derived BMEC-like cells. Hi-PSC marker: POU5F1 (OCT4). Tight junctions: CDH5 (VE-CADHERIN), CLDN5, OCLN, PECAM1 (CD31), TJP1 (ZO-1) and VWF. The qRT-PCR data are plotted as mean ± s.d. Student’s unpaired t-test (****p < 0.0001), *N* = 3. **C**) Schematic representation of transendothelial resistance measurement (TEER). Cells plated on a permeable Transwell™ insert (0.4 μm PET) were assessed for TEER with an EVOM2 stx2 electrode. The diagram was created on Biorender.com **D**) BMEC passive barrier as shown by TEER following subculture for healthy donor hi-PSCs. Error bars represent the standard deviation of triplicate Transwell™ filters (*N* = 3). Statistical significance was determined using One-Way ANOVA (****p < 0.0001). *N* = 3

One of the most challenging aspects of studying the role of the BBB in neurodegenerative diseases is the limited availability of human in vitro models. Hence, we first set off to differentiate brain microvascular endothelial-like cells (BMEC-like cells) from a total of 2 human induced pluripotent stem cell lines (hi-PSCs) derived from healthy donor’s fibroblasts, as previously described by others [19]. As reported, after 7 days of differentiation and subsequent subculture, hi-PSC-derived BMEC-like cells expressed various endothelial markers such as Claudin-5 and vascular endothelial growth factor A (VEGF-A) demonstrated by immunostaining (Fig. 1A). Indeed, upon differentiation, hi-PSCs derived BMEC-like cells showed a transcriptional reduction in the pluripotency marker POU5F1 (OCT4) and showed increased RNA expression of cell-specific genes encoding for tight junction molecules such as Cadherin-5 (CDH5 or Ve-Cadherin), Claudin-5 (CLDN5) and Occludin (OCLN), as well as the platelet endothelial cell adhesion molecule 1 (PECAM1 or CD31) and the Von Willebrand factor (VWF) (Fig. 1B), which are involved in both the establishment and the maintenance of BBB permeability [20].

We then assessed BMEC functionality by measuring transendothelial electrical resistance (TEER) [21]. TEER measurement has been widely used and is the gold standard to assess the functionality of the BBB, both in vivo and in vitro. The maximum TEER recorded in vivo was 5400 Ω x cm^2^, in adult rats [22]. In terms of in vitro modelling, Lippmann and colleagues successfully developed brain endothelial like-cells using human induced pluripotent stem cells, with a TEER of 4000 Ω x cm^2^ [19].

Using a similar approach, BMEC-like cells were seeded onto Transwell™ inserts (Fig. 1C) and monitored daily for 4 days (Fig. 1D). Control BMEC-like cells displayed a typical increase in TEER values over time from day 1 to 3, as tight junctions developed and matured to achieve TEER values above 4000 Ω x cm^2^ at day 3 of subculture across independent differentiations (*N* = 3). As previously reported by others, the resistance rapidly decreased on day 4 [19] (Fig. 1D).

### C9-ALS BMEC-like cells monolayer displays functional abnormalities

**Fig. 2:**
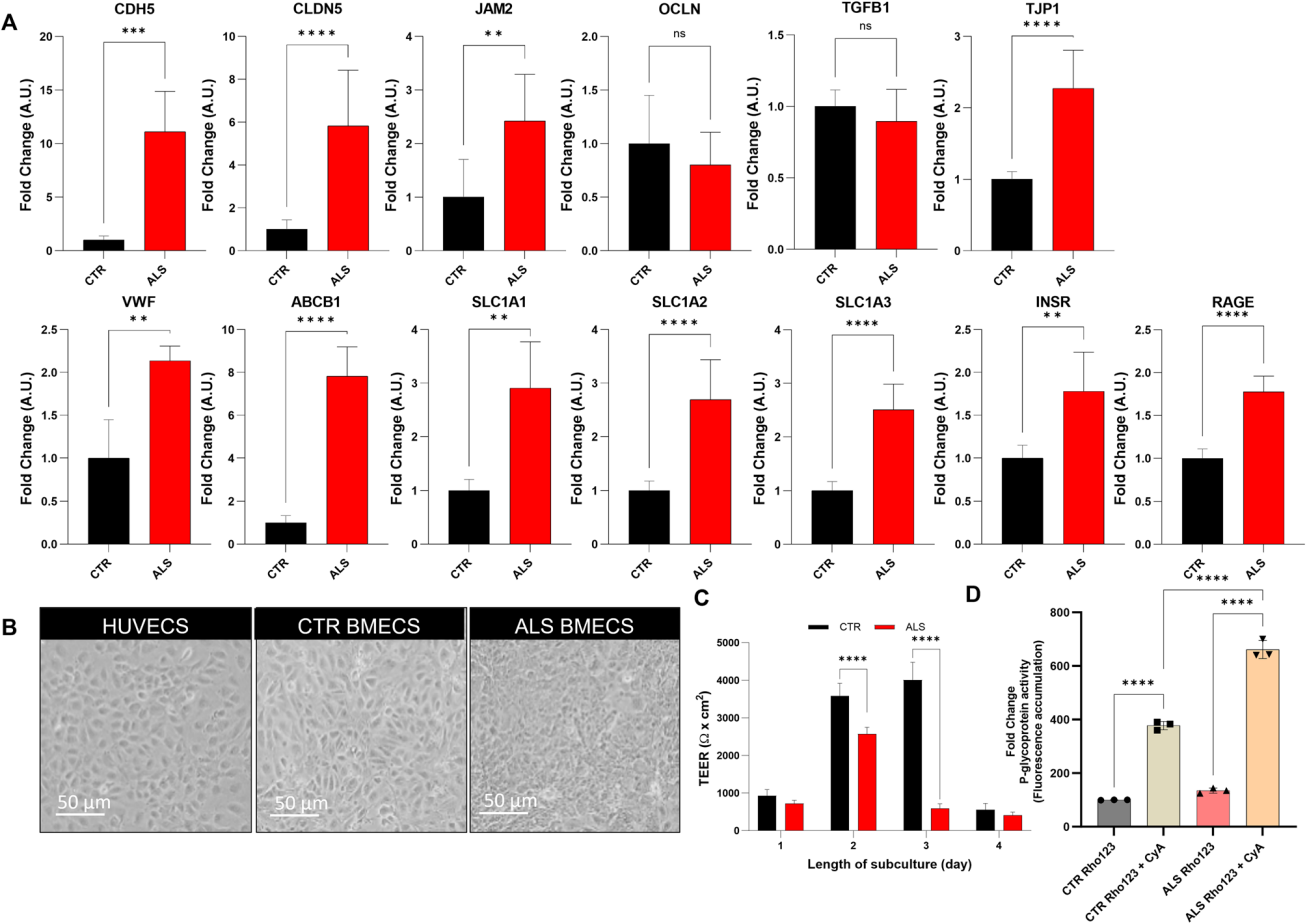
C9-ALS BMEC-like cells form a dysfunctional barrier **A**) Blood-Brain Barrier phenotypic markers. Ve-Cadherin (CDH5), Claudin-5 (CLDN5), Junctional Adhesion Molecule 2 (JAM2), Occludin (OCLN), Transforming growth factor beta 1 (TGFB1), Zonula Occludens 1 (TJP1) and, Von Willebrand factor (VWF), P-glycoprotein (ABCB1), EAAT3 (SLC1A1), EAAT2 (SLC1A2), EAAT1 (SLC1A3), Insulin receptor (INSR) and, receptor for advanced glycation end products (RAGE) transcriptional expression of 2 healthy donors and 3 C9-ALS donors hi-PSCs derived BMEC-like cells. qRT-PCR data are plotted as mean ± s.d. Statistical significance was determined using Student’s unpaired t-test (****p < 0.0001). *N* = 3 per group. **B**) Brightfield images are shown as follows: HUVECs, CTR and C9-ALS BMEC-like cells. The scale bar equals 50 µM. **C**) BMEC passive barrier as shown by TEER following subculture for GM23338 male healthy donor hi-PSCs derived BMEC-like cells (CTR BMECs), CS52iALS-C9nxx and CS29iALS-C9nxx male C9-ALS donors (ALS BMECs). Error bars represent the standard deviation of triplicate Transwell™ filters. Statistical significance was determined using Student’s unpaired t-test (****p < 0.0001). **D**) BMEC-like cells were incubated with or without Cyclosporin-A, a P-glycoprotein inhibitor and, next with Rhodamine-123, a P-glycoprotein fluorescent substrate. Accumulation is normalised to the no-inhibitor samples. Error bars represent the standard deviation of triplicate wells. Statistical significance was determined using One-Way ANOVA (****p < 0.0001). *N* = 3

Confident that the model developed from healthy hi-PSC recapitulated BMEC-like morphological and functional characteristics, we used the same differentiation protocol to interrogate the effects of the *C9ORF72* carriers and two healthy donors of similar age were differentiated into BMEC-like cells (Supplementary Table 1) and were interrogated by RT-qPCR for the expression of several BMEC-related markers that have been previously associated with ALS or BBB dysfunction in other pathologies (Fig. 2A, Supplementary Fig. 1) and immunocytochemistry (Supplementary Fig. 2). Briefly, we quantified transcripts encoding tight junction and adhesion proteins (VE-Cadherin, Claudin-5, JAM-2, Occludin, ZO-1); transporters (ABCB1 and SLC1A1, 2, 3); key receptors (INSR, RAGE); cytokines (TGFB1) and clotting factors (VWF). Overall, excluding Occludin and TGFB1, the data revealed a widespread transcriptional upregulation of BBB-associated structural proteins, transporters and receptors in C9-ALS BMEC-like cells compared to controls (Fig. 2A, Supplementary Fig. 1).

To assess whether these transcriptional alterations were associated with functional dysregulation, the monolayer permeability was tested by TEER measurements. The C9-ALS BMEC-like monolayer appeared visibly intact and macroscopically indistinguishable from its healthy counterpart (Fig. 2B and Supplementary Fig. 3). On day 1 of the assessment, TEER values for control and C9-ALS cells were comparable (1000 and 800 Ω x cm^2^ respectively). However, on day 2, the TEER for C9-ALS BMEC-like cells failed to increase to the same level as the healthy control monolayer, with the C9-ALS BMEC-like TEER averaging approximately 2500 Ω x cm^2^; whereas the control monolayer reached a TEER of 4000 Ω x cm^2^. This significant difference was still present at day 3 with control BMEC-like cells sustaining a TEER measurement of 4000 Ω x cm^2^ and C9-ALS BMCs displaying a loss of membrane electrical resistance (900 Ω x cm^2^). Finally, on day 4, both control and C9-ALS monolayers displayed the typical collapse in TEER measurement [19], which declined below 1000 Ω x cm^2^ (Fig. 2C).

As we had identified a dysregulation in the P-glycoprotein (P-gp) transcript (ABCB1) (Fig. 2B), we next evaluated the efflux activity of this key transporter in BMEC-like cells. P-glycoprotein activity was tested by measuring the accumulation of its substrate, Rhodamine 123, via fluorescence, in the presence or absence of Cyclosporin A, a P-glycoprotein inhibitor (Fig. 2D). Both CTR and C9-ALS BMEC-like cells were incubated with Cyclosporin-A and showed a significant increase in fluorescence accumulation, indicating active P-glycoprotein function. However, patient P-glycoprotein efflux activity was significantly higher than control BMEC-like cells.

### C9-ALS iAstrocytes conditioned medium is toxic to BMEC-like cells

Astrocytes are key players in BBB functionality, as they support BMECs through the secretion of trophic factors and maintain homeostasis. Research carried out by our group and others has shown that astrocytes are major players in ALS pathology [23, 24]. Above all, it has been reported that astrocytes derived from either post-mortem tissues or fibroblasts (iAstrocytes) from ALS patients are toxic towards motor neurons and this toxicity is also transferred through conditioned medium [25, 26]. Hence, we wanted to explore the effect of iAstrocytes on BMEC-like cells.

When C9-ALS BMEC-like cells were co-cultured either with healthy or with C9-ALS iAstrocytes, they displayed very poor TEER properties, comparable to the values obtained from the monocultures (Fig. 1D, Supplementary Fig. 4). Healthy astrocytes could not correct the cell-autonomous defects of the C9-ALS BMEC-like cells and C9-ALS iAstrocytes did not worsen their barrier phenotype (Supplementary Fig. 4).

**Fig. 3:**
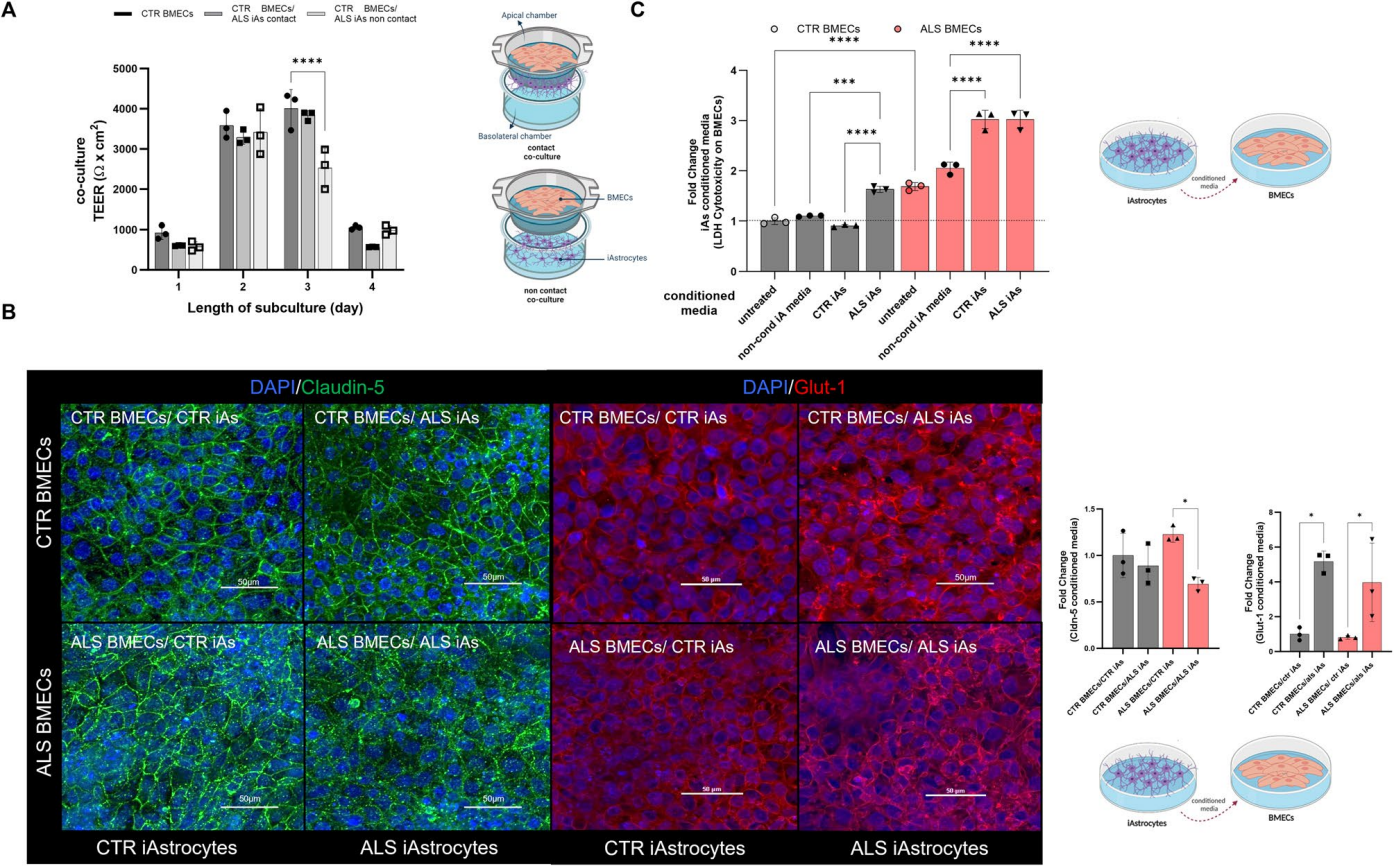
C9-ALS iAstrocytes affect cell dynamics and paracellular permeability on BMEC-like cells **A**) BMEC passive barrier as shown by TEER following BMEC-like cells/iAstrocytes co-culture for C9-ALS BMEC-like cells. Error bars represent the standard deviation of triplicate Transwell™ filters. Statistical significance was determined using One-Way ANOVA (****p < 0.0001). *N* = 3. **B**) BMEC-like cells were either treated with control or C9-iAs-conditioned media. BMEC-like cells were fixed in 100% MeOH after 48 h of the experiment. Scale bar equals 50µM. Plotted results represent confocal image z-stacks analysed in 3D volume. Claudin-5 is shown in green and Glucose-1 transporter (Glut-1) in red; and DAPI in blue for the nuclei staining. Error bars represent the standard deviation of triplicate Transwell™ filters. Statistical significance was determined using One-Way ANOVA (****p < 0.0001). *N* = 3. At least a total of 5 images were acquired per each condition, with a minimum of 3 technical replicates for a total of 3 biological replicates (*N* = 3, total images per condition = 45). **C**) The LDH assay was performed after 2 days of iAstrocytes conditioned media treatment on BMEC-like cells. Untreated refers to BMEC-like cells on their indicated endothelial media and non-cond iA media are BMEC-like cells treated with plain iAstrocytes media to evaluate the effect of the media on the BMEC-like cells. Control (CTR) and C9-ALS media (ALS). Error bars represent the standard deviation of triplicate wells. Statistical significance was determined using One-Way ANOVA (****p < 0.0001). *N* = 3

We next interrogated the effect of C9-ALS iAstrocytes on BMEC-like cells from healthy individuals in a cell-to-cell contact as well as in a non-contact paradigm (Fig. 3A). For this experiment, BMEC-like cells were seeded on the apical chamber of the Transwell™ insert and iAstrocytes were placed either in the basal chamber (contact culture) or in the well underneath, with no physical contact but sharing the culture media (non-contact co-culture). Interestingly, the TEER of healthy BMEC-like cells co-cultured with C9-ALS iAstrocytes in the contact modality was unaffected (Fig. 3A) compared to BMEC-like cells in monoculture, while one would expect an increase in TEER when astrocytes and BMECs are cultured together. In contrast, a decrease in the TEER measurements of 36% (− 1400 Ω x cm^2^) was recorded among the healthy BMEC-like cells when in non-contact co-culture with C9-ALS iAstrocytes.

To further explore the astrocyte-endothelial cells interaction within the barrier features, iAstrocyte conditioned media was added to BMEC-like cells for 48 h. Interestingly, the Glut-1 transporter protein was found to be upregulated in both healthy and C9-ALS BMEC-like cells after adding C9-ALS iAstrocytes conditioned media, thus indicating an active metabolic cross-talk between the two cell types through secreted factors. In contrast, the tight junction Claudin-5 protein expression decreased in the presence of C9-ALS iAstrocyte conditioned media (Fig. 3B). In the most severe condition, C9-ALS BMECs treated with C9-ALS iAstrocyte media, Claudin-5 staining while still junctional, it presents as puncta (Fig. 3B).

Finally, we used the LDH colourimetric assay to determine whether the differences observed in the TEER measurements and protein quantification were due to cytotoxicity. BMEC-like cells from healthy donors were assessed in monoculture at baseline and after 48 h in iAstrocyte conditioned medium **(**Fig. 3C). Plain astrocyte media-treated BMEC-like cells were used as controls (non-cond iA media).

C9-ALS iAstrocyte conditioned medium had a detrimental effect on healthy BMEC-like cells, as shown by an increase in LDH activity of over 60% (Fig. 3C) in comparison to untreated and healthy astrocyte-conditioned medium-treated cells. These results provide evidence that C9-ALS astrocyte toxicity extends beyond motor neurons.

### C9-ALS BMEC-like cells display metabolic defects

Since BMEC-like cells from C9-ALS donors displayed severe functional defects in a cell-autonomous fashion, we next decided to interrogate the expression and localisation of Claudin-5 and Glut-1, the major tight junction and main glucose transporter of the BBB respectively.

At the transcriptional level, C9-ALS BMEC-like cells displayed upregulation of CLDN5 (Claudin-5) (Fig. 1A), however, protein quantification and localisation were comparable to their healthy counterparts (Supplementary Fig. 2).

**Fig. 4:**
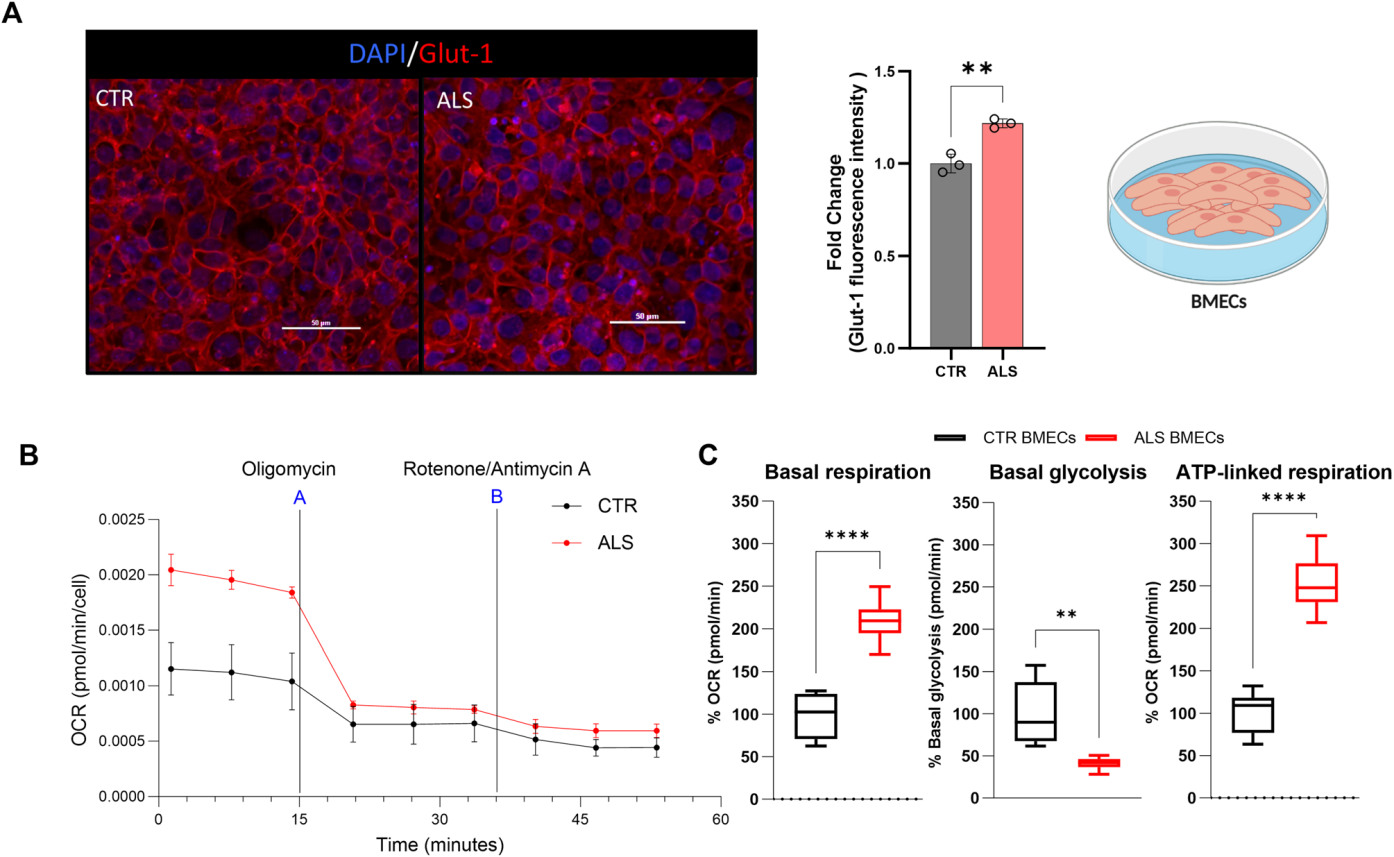
C9-ALS BMEC-like cells have an increased glucose metabolism and altered mitochondrial respiration **A**) Healthy donor hi-PSCs derived BMEC-like cells (CTR BMECs) and C9-ALS donor hi-PSCs derived BMEC-like cells (ALS BMECs) confocal images. BMEC-like cells were fixed in 100% MeOH. Scale bar equals 50µM. Images and results represent confocal z-stacks analysed in 3D volume. Glucose-1 transporter (Glut-1) in red and DAPI in blue for the nuclei staining. Error bars represent the standard deviation of triplicate Transwell™ filters. Statistical significance was determined using Student’s unpaired t-test (****p < 0.0001). At least a total of 5 images were acquired per each condition, with a minimum of 3 technical replicates for a total of 3 biological replicates (*N* = 3, total images per condition = 45). **B**) Mitochondrial Real-Time ATP rate test was carried out on healthy donor hi-PSCs derived BMEC-like cells (CTR BMEC-like cells), and 3 C9-ALS donors hi-PSCs derived BMEC-like cells (ALS BMEC-like cells). Data were acquired and analysed using the Agilent Technologies Sea-horse platform and software. Oxygen consumption rate (OCR) measurement per cells is represented as a time curse. The addition of the mitochondrial drugs Olygomycin (A) and Rotenone/Antimycin A (B) are indicated in the graph. **C**) Mitochondrial Real-Time ATP rate test was carried out on healthy donor hi-PSCs derived BMEC-like cells (CTR BMEC-like cells), and 3 C9-ALS donors hi-PSCs derived BMEC-like cells (ALS BMEC-like cells). Data were acquired and analysed using the Agilent Technologies Sea-horse platform and software. Error bars represent the standard deviation of triplicate wells *N* = 3. Data is plotted as Min-Max Box and Whisker. Statistical significance was determined using Student’s unpaired t-test (****p < 0.0001)

However, the expression of Glut-1 was significantly altered in C9-ALS BMEC-like cells, compared to controls, with an overall increase in protein immunofluorescence signal (Fig. 4A).

Due to the important metabolic role of BMECs at the BBB and the identified dysregulation in Glut-1, we decided to assess the metabolic function of healthy and C9-ALS BMEC-like cells using the Seahorse XF Real-Time ATP Rate Assay in the presence of mitochondria inhibitors (Fig. 4B). Consistent with the hypothesis that Glut-1 dysregulation would have downstream effects on metabolism; the data showed that C9-ALS BMEC-like cells display significantly higher basal respiration and ATP-linked respiration, while basal glycolysis is significantly downregulated (Fig. 4C).

### C9-ALS BMEC-like cells conditioned medium reduce neurite length of healthy MNs

**Fig. 5:**
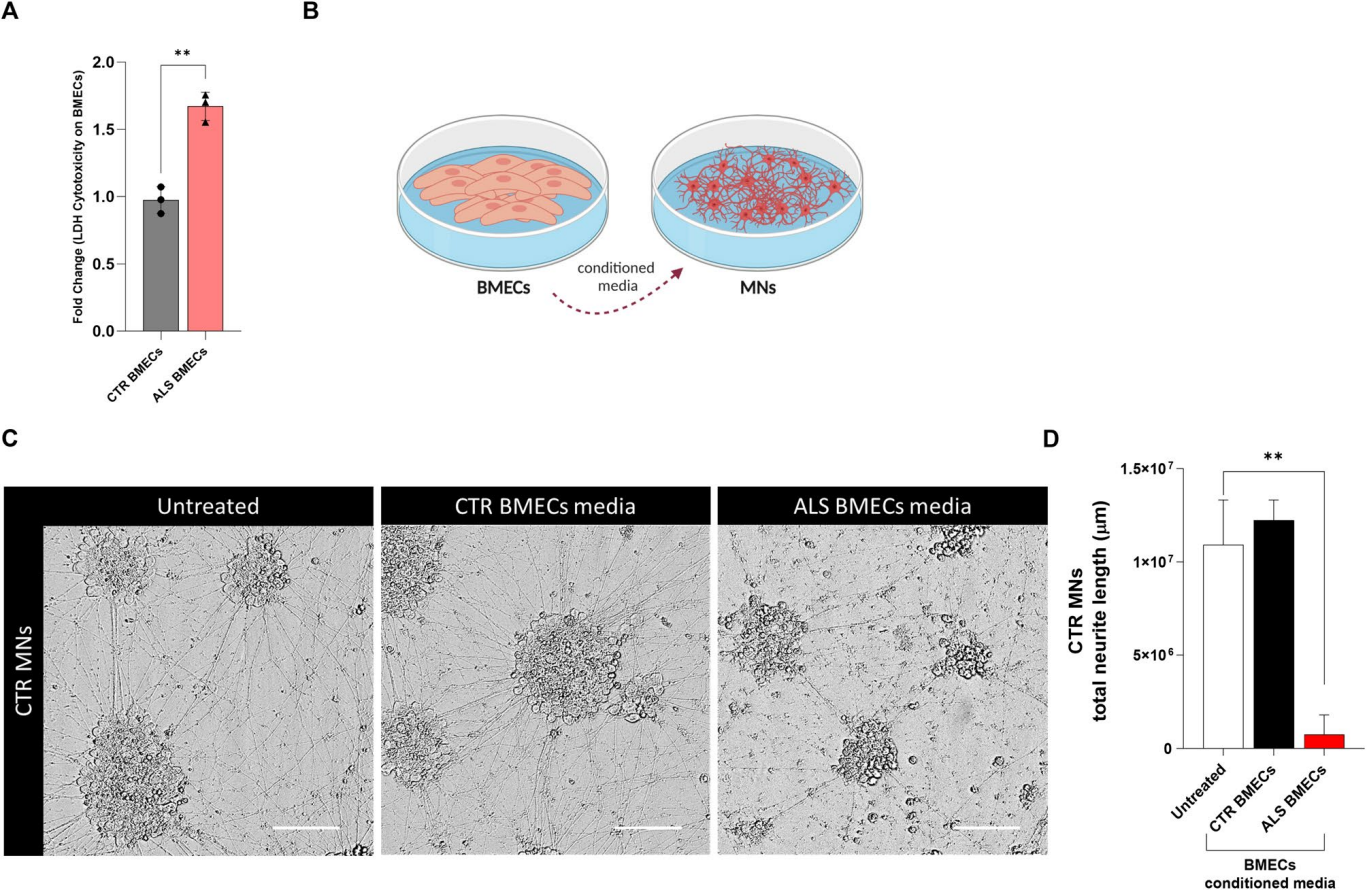
C9-ALS BMEC-like cells restrict neurite length in healthy Motor Neurons **A**) The Lactate Dehydrogenase Assay (LDH) assay was performed on 2 healthy donors and 3 C9-ALS donors hi-PSCs derived BMEC-like cells. Statistical significance was determined using Student’s unpaired t-test (****p < 0.0001). **B**) The diagram was created on Biorender.com **C**) Control Motor Neurons (MNs) were fixed with 4%PFA after 72 h on 30% BMEC-like cells conditioned media treatment. Brightfield images are shown as follows: untreated MNs, control BMEC-like cells media (CTR BMECs) and ALS BMEC-like cells media (ALS BMECs) treated MNs. The scale bar equals 50 µM. At least a total of 5 images were acquired per each condition, with a media of 3 replicates for a total of 3 biological replicates (*N* = 3, total images per condition = 45). **D**) Neurite length analysis was performed with ImageJ software. Data are plotted as Mean ± SD. Statistical significance was determined using One-Way ANOVA (****p < 0.0001). *N* = 5

Once established that C9-ALS BMEC-like cells display cell-autonomous defects, such as impaired TEER and metabolism along with mild levels of cytotoxicity, we decided to explore the effect of BMEC-like cells on healthy control (CTR) motor neurons (MNs). To avoid confounding effects from contact cultures, we used the BMEC-like cells conditioned media, shown to be cytotoxic as per an increase in the lactate release, measured by the LDH test (Fig. 5A). MNs were derived from healthy hi-PSCs and treated with 30% BMEC-like cells conditioned medium for 3 days (Fig. 5B). No differences among MNs treated with CTR BMEC-like cells conditioned media or untreated were observed. Hence, contrary to what is reported for astrocytes [27], the BMEC-like cells conditioned media from healthy donors did not improve neurite length in healthy MNs (Fig. 5C, D). However, C9-ALS BMEC-like cells conditioned medium significantly decreased the total neurite length of treated CTR MNs by 93.8% (Fig. 5C and D), hence causing extensive cell death.

## Discussion

The BBB is a multi-functional compartment that shields the brain from external agents to protect it from infections and toxic molecules. It facilitates gas and nutrient exchange through a complex network of endothelial cells, astrocyte end-feet, and pericytes along capillaries.

BBB dysfunction is implicated in several neurodegenerative disorders [3]. While a clearer link has been described in dementia [28], little is known about the role of endothelial cells in ALS pathogenesis [29].

Given that *C9ORF72* (C9) repeat expansions are the most common genetic cause of ALS and frontotemporal dementia [12], we decided to investigate the characteristics and function of brain microvascular endothelial cells (BMEC-like cells) using 5 different C9-ALS patient-derived pluripotent stem cell lines (hi-PSCs).

First, we assessed the endothelial properties of BMEC-like cells. In contrast to the results reported in C9-ALS animal models, where most of the tight junctions seem to be downregulated at the protein level [16]; many of the transcripts were upregulated in C9-ALS BMEC-like cells. An example is Cadherin-5 (Ve-Cadherin), which is exclusively expressed in endothelial cells and is involved in adherens junction assembly and maintenance [30, 31]. The junctional adhesion molecule Jam-2 (Jam-b) is involved in the regulation of cell polarity, endothelium permeability and leukocyte migration. Jam-2 downregulation might have a positive effect on immune disorders, suggesting a role in disease pathophysiology [32]. The tight junction 1 or ZO-1 transcript level was also higher in C9-ALS BMEC-like cells compared to CTR-BMEC-like cells. ZO-1 is essential for BBB assembly, as this protein is responsible for the organization of various components of the tight junction and for linking them to cytoskeletal actin [33].

Gene expression alterations do not always correspond to alterations in protein levels [34] due to the several processing steps between RNA and protein synthesis, especially in disease cases, such as C9-ALS, where RNA nucleo-cytoplasmic transport, as well as protein synthesis and turnover, are impaired [35].

In our study, Claudin-5 and other tight junctions were significantly upregulated at the mRNA level in C9-ALS BMEC-like cell monocultures compared to healthy BMECs, while protein levels in the membrane appeared unchanged via immunostaining. These results are inconsistent with the significant barrier defects detected via TEER measurements. In this context, transcriptional upregulation might reflect a compensatory mechanism to counteract a functional defect and failure to detect protein expression alterations could be explained by the technical limitations of immunostaining, which is only semi-quantitative and might fail to detect small changes. In addition, staining quantification was performed on the whole cell, rather than in the membrane, where tight junctions fulfil their function, hence potentially accounting for the difference between protein expression and functionality.

Of note, BBB integrity is not only linked to Claudin-5, in fact, several Claudins contribute to BBB electrical resistance [36]. Hence, our assessment of specific causative tight junction changes responsible for membrane permeability defects is limited. Several factors, such as an overall dysregulation of tight junction expression and membrane composition, post-translational modifications and splicing variants could cause defects in C9-ALS BMEC barrier properties. Subtle changes in BBB permeability can also be detected via tracer permeability assays, such as fluorescein and Evans blue-albumin, which were not performed in this study.

Lactate dehydrogenase (LDH) cytotoxicity or release test, which measures the LDH in the cell culture supernatant showed that C9-ALS BMEC-like cells showed a cytotoxicity value of over 50% compared to the healthy control cells. Although functional TEER defects could also be explained by the reported increased levels of cytotoxicity detected in C9-ALS BMEC-like cells, we have not observed macroscopic levels of cell loss in the cultures, as indicated by the Eosin Y staining or differences in cell numbers assessed during staining. It cannot be excluded, however, that cell death might occur over the 4-day assay during which TEER was measured.

The defects we have highlighted in this study, however, cannot be solely linked to cell death. C9-ALS BMEC-like cells displayed a significant increase in the expression and function of the P-glycoprotein transporter (ABCB1), which is crucial for drug delivery. P-glycoprotein upregulation is a very common feature in many diseases, including ALS, epilepsy and cancer in in vivo models [37, 38]. It is of great relevance for the development of disease-specific drug-permeability in vitro assays that the BBB model described here recapitulates increased P-gp functionality, thus providing a unique tool for the advancement of such pharmacodynamic assays.

Another membrane protein crucial for endothelial cell function and its relationship with neurons is Glut-1, the main glucose transporter expressed in the BBB. Our data highlighted increased expression of this protein in C9-ALS BMEC-like cells, with a concomitant severe decrease in basal glycolysis and a potentially compensatory increase in mitochondrial respiration. Recently, Kim et al. reported that brain endothelial cells are preferentially glycolytic, and this is associated with the maintenance and permeability of the BBB [39]. Kim and colleagues showed an impaired endothelial barrier permeability to molecules utilizing the transcellular pathway upon glycolysis inhibition [39]. Glycolysis therefore has a vital role in BBB homeostasis, promoting vessel branching and modulating angiogenesis [40].

Interestingly, this is dissimilar to the metabolic defects reported in other models of *C9ORF72* pathology, where neurons have mostly displayed defects in mitochondrial respiration [42]. Thus, while endothelial cells are preferentially glycolytic [41], motor neurons mostly use mitochondrial respiration to produce energy [42].

Our previous research on C9-ALS patient-derived astrocytes (iAstrocytes) demonstrated that *C9ORF72* astrocytes affect neuronal networks through cell-to-cell contact [25], as well as through the secretion of toxic factors [27]. Consequently, we decided to explore the effect of iAstrocytes on BMEC-like cells, given the crucial role of astrocytes in BBB homeostasis.

Consistent with the toxic properties of astrocytes towards motor neurons, C9-ALS iAstrocytes caused severe impairment of the TEER properties of healthy BMEC-like cells both through contact and conditioned medium, thus indicating that secreted factors might be the culprit. In fact, C9-ALS iAstrocyte conditioned medium treatment caused an upregulation in the Glut-1 transporter in both control and C9-ALS BMEC-like cells; while it caused a decrease in Claudin-5 expression in ALS BMECs. Although C9-ALS astrocytes only induced a decrease in TEER in non-contact cultures, the lack of an increase in TEER in the contact cultures with BMECs and C9-ALS astrocytes compared to BMECs only is unexpected. In fact, the electrical resistance of combined BMECs and astrocytes should be higher than BMECs on their own, thus suggesting that C9-ALS astrocytes affect healthy BMECs in both paradigms tested. Interestingly, C9-ALS iAstrocytes only mildly increased the already existing defects in C9-ALS BMEC-like cells, indicating that these cells are affected by the presence of mutations in *C9ORF72* in a cell-autonomous fashion.

Indeed, BMEC-like cells not only are intrinsically affected by the *C9ORF72* mutation, but they also display highly toxic properties against healthy human motor neurons via secreted factors, similar to astrocytes. C9-ALS BMEC-like conditioned medium treatment significantly increased cell death and subsequently reduced motor neuron neurite length by more than 90% after only 3 days of exposure. As previously reported, neurotoxicity is not a property common to any cell type derived from ALS patients, in fact, fibroblasts do not display such characteristics [25].

Although genetic experiments in in vivo models of SOD1-ALS had led to the conclusion that endothelial cells played no role in disease pathogenesis [43], our data demonstrate that C9-ALS endothelial cells are intrinsically affected by the presence of the *C9ORF72* mutation. These cells display hyperactivation of P-gP transporters, which can cause pharmaco-resistance; and alterations in tight junction expression together with associated membrane permeability [44]. In addition, C9-ALS BMECs also displayed a marked decrease in glycolysis alongside an increase in mitochondrial energy production, thus indicating a metabolic shift that can affect membrane permeability [45] and substrate exchange with neurons [46]. Most importantly, our data demonstrate that C9-ALS BMECs can play a significant role in non-cell autonomous motorneuronal death through secreted factors.

## Materials and methods

### Cell culture

#### Human umbilical vein endothelial cells (HUVECs)

Cells were purchased from ATCC (CRL-1730) and routinely seeded on uncoated flasks or 6-well plates. Following the manufacturer’s recommendations, Human Endothelial Serum Free Medium (hESFM, Gibco), supplemented with 10% Foetal Bovine Serum (FBS, Gibco) and 1% penicillin/streptomycin media was replaced every 24-48 h, when cells reached 70% confluence. Cells were split when they approached 85% confluence by removing the media and washing twice with HBSS with no calcium/magnesium. Then, cells were incubated with trypsin for up to 10 min at 37 °C until the cells became completely round. Afterwards, the cell suspension was collected and subsequently centrifuged for 7 min / 100 g. Finally, the cell pellet was homogenised in 1 ml of fresh media, cells were counted with an automatic haemocytometer by mixing 10 µl of cell suspension with 10 µl of trypan blue and seeded following the supplier recommendations (2.3 × 10^3^ viable cells / cm^2^).

#### Human induced pluripotent stem cells derived brain endothelial-like cells

Brain endothelial-like cells (BMEC-like cells) were generated by culturing human induced pluripotent stem cells (hi-PSCs) in mTeSR plus media (STEMCELL Technologies) on Matrigel-coated (Corning) plates as previously described by Lippmann and Neal et al. protocols [19, 47, 48]. When hi-iPSCs reached more than 80% of confluence, they were detached by incubating the plate with accutase, for 5 min at 37 °C. Next, cells were collected and centrifuged for 4 min / 200 g and resuspended in TeSR™-E8™ media (STEMCELL Technologies). Subsequently, cells were counted with an automatic hemocytometer as described before and seeded at appropriate density (∼ 14 × 10^3^ viable cells x cm^2^) in matrigel coated 6w- plates.

Day 1 of differentiation began two days after seeding, or when cells reached 60–70% confluency, by switching the media to serum-free media TeSR™-E6™ (STEMCELL Technologies), refreshing it daily for 4 days. On day 5, during the expansion phase, the media was changed to EM^+^ (hESFM supplemented with 10 µM Retinoic Acid (RA, Sigma), 20 ng/mL bFGF (STEMCELL Technologies), 0.5% B-27 (Gibco), and maintained for 48 h). At day 7 of differentiation, cells were subcultured. Firstly, the media was removed, and cells were washed with HBSS with no calcium/magnesium and 1 ml of accutase (STEMCELL Technologies) was added per well and incubated at 37 °C 20–45 min until a single cell suspension was formed. Then, cells were gently collected and centrifuged for 4 min/ 200 g. Optionally, cells can be stored in liquid nitrogen with 10% DMSO (Sigma). Finally, cells were reseeded or thawed in a mix of collagen IV and fibronectin pre-coated plates or cell culture inserts and maintained for one day in EM^+^ plus ROCK inhibitor (Tocris) at a final density of 1 million cells/ cm^2^. At day 8, media was switched to hESFM supplemented with 0.5% B-27 only until the end point. At day 9 BMEC-like cells are ready to be tested.

Cell lines’ relevant clinical information is described in Supplementary Table 1.

#### Hi-PSCs-derived motor neurons

Neural differentiation was performed using a modified version of the dual SMAD inhibition protocol [49]. Briefly, hi-PSCs in the presence of small molecules: ROCK inhibitor, SMAD inhibitors (SB431542 and DMH1) for 6 days, SB431542 (Tocris), DMH1 (Tocris), RA and Purmorphamine (Tocris) for another 6 days. At that point, all the hi-PSC lines generated more than 90% OLIG2 + Motor Neuron Progenitor Cells. Afterwards, the cells were expanded in the same media, supplemented with valproic acid (STEMCELL Technologies).

To induce MN differentiation, OLIG2 + MNPs were dissociated and cultured at a density of 1:6 in suspension in neural medium with RA and Purmorphamine. The medium was changed every other day for 6 days. Next, they were dissociated into single cells and plated on Matrigel-coated plates and cultured with RA, Purmorphamine and Compound E (γ-Secretase-IN-1, Sigma) for 10 days, after that, the media was replaced with neuronal media (neurobasal media supplemented with 1% of B27, BDNF 10ng/mL, CNTF 10ng/mL and IGF 10ng/mL). The cells were then fed on alternate days with neuronal medium until day 40 to mature into CHAT + MNs.

Cell lines relevant clinical information is described in Supplementary Table 1.

#### Hi-NPCs derived iAstrocytes

Fibroblasts were reprogrammed to human-induced neuronal progenitor stem cells (hi-NPCs) following the Meyer et al. 2014 protocol [25]. In brief, fibroblasts were seeded in a well of a six-well plate and treated with a mixture of retroviral vectors expressing Kruppel-like factor 4 (Klf4), POU transcription factor Oct-3/4 (Oct3/4), SRY-related HMG-Box Gene 2 (Sox2), and c-Myc for 12 h (System Biosciences). To promote neuroprogenitor cell conversion, the culture medium was switched 72 h after to a medium containing bFGF, epidermal growth factor (EGF, STEMCELL Technologies), and heparin (Sigma), and this was continued for 18 days.

To obtain iAstrocytes, hi-NPCs were seeded in 2.5 µg/ml fibronectin-coated 10 cm dishes onto Knockout DMEM-Ham’s F12 (Gibco), GlutaMAX supplement (STEMCELL Technologies); plus 10% FBS, 1.8% 100x diluted N2 (ThermoFisher) and 1% Penicillin/Streptomycin (Sigma); according to Meyer and Ferraiuolo’s protocol [25], for 7 days.

Cell lines relevant clinical information is described in Supplementary Tables 1 and 2.

#### BMEC-like cells and iAstrocytes conditioned media and co-culture

At day 7 of BMEC-like cell differentiation cells were subcultured and seeded on the upper part of cell trans-well inserts (0.4µM, PET, Corning) or in 96-well clear bottom black (Fisher) at a density of 1 million cells/cm^2^. At day 8, BMEC-like cells were co-cultured with mature iAstrocytes. If performing conditioned media experiments, iAstrocytes conditioned media was collected and fast-frozen using dry ice. For co-culture, the iAstrocytes in dishes were washed once with PBS and detached by adding accutase at 37 °C. Then, the iAstrocyte suspension was collected and centrifuged for 4 min/200 g. At that time, iAstrocytes were replated either at the bottom of the 2.5 µg/ml fibronectin-coated trans-well insert and incubated for 1 h at 37 °C (contact co-culture) or in the well below the Transwell™ insert (non- contact co-culture) at a density of 0.3 cells/cm^2^. Finally, conditioned media and cells were tested after 2 or 3 days.

### Barrier tightness and transport

#### Transendothelial resistance (TEER) measurements

BMEC-like cells were seeded in fibronectin/collagen (Corning/Sigma) 0.4 μm PET trans-well inserts when subculturing, as previously described. TEER (transendothelial resistance) measurements were monitored daily after day 8 of differentiation (day 1 of subculture), with an EVOM2 stx2 electrode. Before the TEER measurements, the cell’s monolayer was visually checked, and only intact monolayers were used. Three measurements per insert were recorded at a standard time each day by placing the electrode chopsticks on the Transwell™ inserts containing the cells. One plate was recorded at a time to avoid artificially raising TEER because of having the plate a prolonged time outside the incubator as suggested by Stebbins et al. [50]. Finally, the plotted values were normalized by subtracting the blank (TEER from an empty insert) and then multiplied by the surface area (0.33 cm^2^) of the transwell filter and reported as Ω × cm^2^.

#### Efflux activity: P-glycoprotein transporter

Similarly, BMEC-like cells are tested on day 9 of the differentiation protocol. For this assay, cells were seeded in a 24-w plate at day 7. Later, at day 9, media was aspirated, and cells were washed with HBSS with no calcium/magnesium. Next, 3 wells per condition were incubated for 1 h at 37 °C with HBSS only and 3 wells with HBSS + 10µM Cyclosporin A (CyA, R&D Systems), a P-glycoprotein inhibitor to inhibit transport. Then, the solution was aspirated, and the cells were incubated for 1 h at 37 °C with HBSS + 10µM Rhodamine 123 (ThermoFisher), a P-glycoprotein substrate; or with HBSS + Rhodamine 123 + CyA respectively. Afterwards, BMEC-like cells were rinsed with PBS twice and fluorescence was measured at Ex 488/ Em 530 nm with a PHERAstar® high-throughput screening microplate reader.

### Transcription quantification by qRT-PCR

Cells were collected and washed twice with PBS and centrifuged for 4 min / 200 g subsequently. RNA was extracted following the manufacturer’s instructions using the RNeasyPlus MiniKit (Qiagen). The RNA concentration was determined with a NanoDrop and the RNA to cDNA reverse transcription was performed following the manufacturer indications for the High-Capacity cDNA Reverse Transcription Kit (ThermoFisher). The RNA samples were standardised to 100 ng/µl. The qRT-PCR was finally completed by adding 50 ng of DNA per well in 384 well plates. The total volume per well was 10 µl, containing 50% SYBR Green, 5µM 2.5% forward Primer, 5µM 2.5% reverse Primer (Supplementary Table 3), 5% sterile ultra-pure water and 40% of cDNA. Transcripts were quantified by calculating the ratio between the expression of each transcript against the housekeeping gene and normalizing the expression to the reference control cell line, i.e., CTR hi-PSC in Fig. 1B or CTR BMEC-like cells in Fig. 2A.

### Protein quantification by immunocytochemistry

Cells were washed twice with PBS and fixed either with 4% paraformaldehyde (ThermoFisher) for 20 min or 100% cold methanol for 10 min. Subsequently, the cells were incubated for 1 h at RT with a blocking solution of PBS, 5% animal serum and 0.3% Triton for non-membrane epitopes. Next, cells were rinsed 3 times with PBS. Then, they were incubated with the primary antibody at 4 °C overnight (Supplementary Table 3). The following day, cells were rinsed 3 times with PBS and incubated for 1 h at RT with the secondary antibody (Supplementary Table 3). Nuclei were stained with 1 µg/ml Hoechst 33342 for 5 min. Finally, the cells were washed 3 times with PBS and the images were normally acquired with a Nikon confocal microscope or with the Opera Phenix™ high-content screening system microscope.

### Fluorescence image acquisition

Z-stack images were captured using an AX R confocal on a Ti2-E base with an LUA-S4 laser launch and a DUX-VB 4-channel GaAsP detector (Nikon Instruments). The images were captured as z-stacks in NIS-Elements AR software (Nikon Instruments) with a 0.5-µm step size in 2 K resonant mode using the 20x Plan Apochromat Lambda D objective (Nikon Instruments) and a 1AU pinhole for a final lateral resolution of 0.17 μm/pixel. For each experiment, at least a total of 5 images were acquired per condition, with a minimum of 3 technical replicates for each of the 3 biological replicates (*N* = 3, total images per condition = 45).

### Fluorescence images analysis

NIS-Elements software (Nikon Instruments, v. 5.42) with the general analysis 3 (GA3) module was used to process and analyse images in 3D volume format. All images were pre-processed with Denoise.ai to remove confocal shot noise and facilitate segmentation. For each z-stack, an automatic threshold was calculated from the background signal intensity for each channel and was used to segment the volume that contained cells stained for the marker of interest. The total cell volume and the mean signal intensity in that volume were automatically measured, along with the mean signal intensity in the volume that did not contain cells (i.e., background intensity). The final reported mean signal intensity was corrected by subtracting the background intensity from the measured mean.

### Cytotoxicity assay

To measure the cellular toxicity within the cells, a lactate dehydrogenase (LDH) assay was assessed following the manufacturer’s indications (CyQUANT™, ThermoFisher). BMEC-like cells were seeded at appropriate density in a 96-w plate at day 7 of the differentiation protocol. After 2 days, the LDH test was done, and the absorbance was measured at 490/680 nm with a PHERAstar® high-throughput screening microplate reader. To determine LDH activity, 682 nm absorbance was subtracted from the 490 nm signal. Lysed cells were considered the maximum LDH activity control and media without cells was used as a blank.

### Mitochondrial kinetics test

To test the effect of cell types on each other’s function, BMEC-like cells were seeded at the desired density in a 96-w Agilent assay plate. The Agilent Seahorse XF Real-Time ATP Rate Assay evaluates key parameters of mitochondrial function by directly measuring the oxygen consumption rate (OCR) of cells on the Seahorse Analysers. It is a plate-based live cell assay that allows the monitoring of OCR in real-time. Non-mitochondrial respiration, which is the oxygen consumption that persists after the addition of rotenone and antimycin A (complex I and III electron transport chain inhibitors respectively) is used to obtain an accurate measure of true mitochondrial respiration. On the other hand, basal respiration which is being used to drive ATP production is measured upon injection of oligomycin (ATP synthase inhibitor) showing the ATP produced by the mitochondria.

### Neurite length quantification

After 3 days of BMEC-like cells conditioned media treatment, motor neurons were fixed with 4% paraformaldehyde and brightfield images per were acquired with the Opera Phenix™ high-content screening system microscope. In addition, ImageJ software was used for complementary image processing and neurite length quantification [51].

### Data Analysis

All statistical analyses were undertaken with GraphPad Prism software (v.10); details of the statistical analyses have been indicated in each figure legend. Immunocytochemistry images were processed with either Columbus™ Image Data Storage and Analysis system software or Nikon NIS-Elements software (v. 5.42).

### Electronic supplementary material

Below is the link to the electronic supplementary material.

Supplementary Material 1

Supplementary Material 2

## Data Availability

The datasets used and/or analyzed are included in this manuscript and all materials are commercially available.
