# Supplementary material for: C9ORF72 patient-derived endothelial cells drive blood-brain barrier disruption and contribute to neurotoxicity
Source: Fluids Barriers CNS. 2024 Apr 11;21:34. doi: 10.1186/s12987-024-00528-6 (PMC11007886; doi:10.1186/s12987-024-00528-6)
Supplement: Supplementary file 1 — Supplementary Material 1 [file 12987_2024_528_MOESM1_ESM.docx]

**Supplementary data**

Supplementary Figure 1

Blood-Brain Barrier phenotypic markers. Ve-Cadherin (CDH5), Claudin-5 (CLDN5), Junctional Adhesion Molecule 2 (JAM2), Occludin (OCLN), Transforming growth factor beta 1 (TGFB1), Zonula Occludens 1 (TJP1) and, Von Willebrand factor (VWF), P-glycoprotein (ABCB1), EAAT3 (SLC1A1), EAAT2 (SLC1A2), EAAT1 (SLC1A3), Insulin receptor (INSR) and, receptor for advanced glycation end products (RAGE) relative transcriptional expression to the housekeeping of 2 healthy donors and 3 C9-ALS donors hi-PSCs derived BMEC-like cells. qRT-PCR data are plotted as mean ± SEM. N=3 per group.

Supplementary Figure 2

Immunocytochemistry of BMEC cells differentiated from a healthy hi-PSCs donor total fluorescence intensity quantification. Claudin-5, Pecam-1 (CD31) and Vegf expression using the RA-enhanced Lippmann’s laboratory protocol from 2019^23^. Scale bar 50um. N=3.

Claudin-5 and VEGF A images were acquired with a Nikon confocal microscope. In contrast, PECAM-1 was acquired with the Opera Phenix™ high-content screening system microscope. As a result, PECAM-1 images are not maximum projection and the 3D nature of the BMECs might look as gaps in the monolayer.

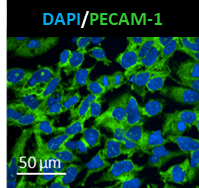


**Supplementary Figure 3**

BMEC barrier integrity was visually analysed by Eosin staining. Control and C9-ALS BMEC-like cells were stained with Eosin-Y and Hoechst solution for 1 and 5 minutes respectively. Brightfield images and EVOS fluorescence images (Eosin Y, Hoechst and combined) are shown. The scale bar equals 150 and 100 µM for the brightfield and fluorescence images respectively.


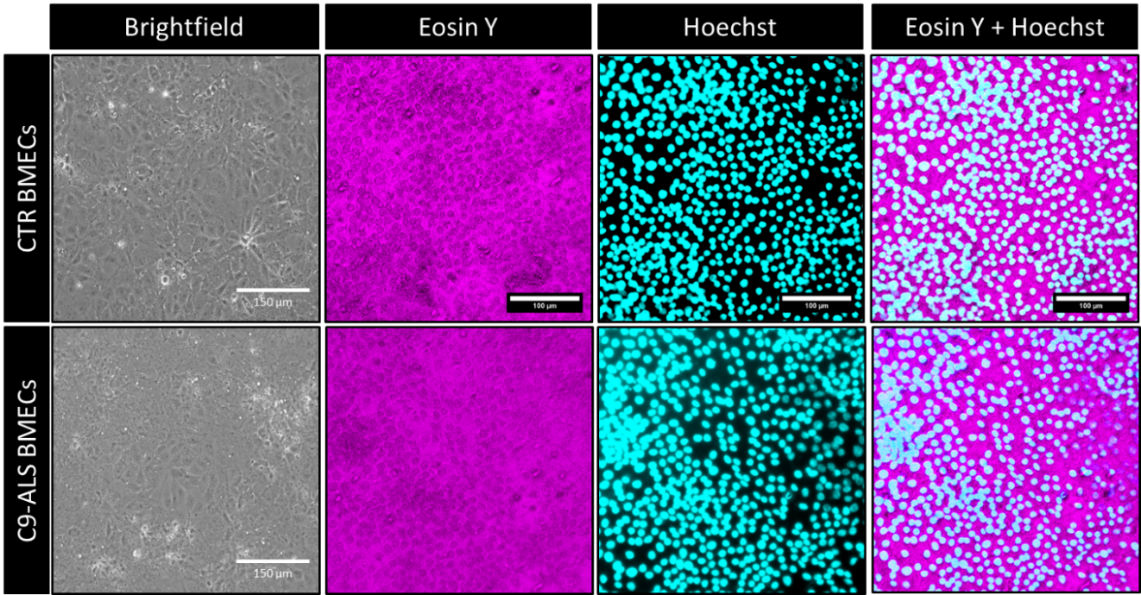


**Supplementary Figure 4**

BMEC passive barrier as shown by TEER following BMEC-like cells/iAstrocytes co-culture for C9-ALS BMEC-like cells. Error bars represent the standard deviation of triplicate Transwell™ filters. Statistical significance was determined using Two-Way ANOVA (****p < 0.0001). N=3.
