# Supplementary material for: C9ORF72 patient-derived endothelial cells drive blood-brain barrier disruption and contribute to neurotoxicity
Source: Fluids Barriers CNS. 2024 Apr 11;21:34. doi: 10.1186/s12987-024-00528-6 (PMC11007886; doi:10.1186/s12987-024-00528-6)
Supplement: Supplementary file 2 — Supplementary Material 2 [file 12987_2024_528_MOESM2_ESM.docx]

**Supplementary Table 1**

Hi-PSCs cell lines used for this research. All cell lines source tissue is fibroblasts.

(*)Cells available either as hi-PSCs or hi-NPCs.

| Cell line | **Supplier** | Reprogramming Method | **Clinical** | **Mutation protein** | **Ethnicity** | **Gender** | **Age at sampling** |
| --- | --- | --- | --- | --- | --- | --- | --- |
| GM23338 | Coriell Insitute | Retrovirus | **Control** | None | Caucasian | Male | 55 years |
| CS14iCTR-21nxx* | Cedars Sinai | Episomal Plasmid | **Control** | None | Unknown | Female | 52 years |
|  |  | Retrovirus |  |  |  |  |  |
| CS52iALS-C9nxx* | Cedars-Sinai | Episomal Plasmid | **ALS** | *C9ORF72* | Caucasian | Male | 49 years |
|  |  | Retrovirus |  |  |  |  |  |
| CS29iALS-C9nxx* | Cedars-Sinai | Episomal Plasmid | **ALS** | *C9ORF72* | Caucasian | Male | 47 years |
|  |  | Retrovirus |  |  |  |  |  |
| CS28iALS-C9nxx* | Cedars-Sinai | Episomal Plasmid | **ALS** | *C9ORF72* | Caucasian | Male | 47 years |
| ALS-183* | University of Sheffield | Sendai Virus | **ALS** | *C9ORF72* | Caucasian | Male | 50 years |
|  |  |  |  |  |  |  |  |
| ALS-78* | University of Sheffield | Sendai Virus | **ALS** | *C9ORF72* | Caucasian | Male | 66 years |
|  |  |  |  |  |  |  |  |

**Supplementary Table 2**

Hi-NPCs cell lines used for this research. All cell lines source tissue is fibroblasts.

(NCH): Nationwide Children’s Hospital, Columbus, OH

| Cell line | **Supplier** | Reprogramming Method | **Clinical** | **Mutation protein** | **Ethnicity** | **Gender** | **Age at sampling** |
| --- | --- | --- | --- | --- | --- | --- | --- |
| 161 | NCH | Retrovirus | **Control** | None | Caucasian | Male | 31 years |
| AG8620 | NCH | Retrovirus | **Control** | None | Caucasian | Female | 64 years |
| 155 | NCH | Retrovirus | **Control** | None | Caucasian | Male | 40 years |
| S3 | NCH | Retrovirus | **Control** | None | Asian | Male | Unknown |
| ZKW542 | NCH | Retrovirus | **Control** | None | Unknown | Female | 8 years |

**Supplementary Table 3**

Primers used for qRT-PCR. Oligos designed and purchased from ThermoFisher.

| Gene  Name | Gene  ID | Forward Sequence (5’-) | Reverse Sequence (5’-) |
| --- | --- | --- | --- |
| ABCB1 | 5243 | TGAATCTGGAGGAAGACATGAC | CCAGGCACCAAAATGAAACC |
| CDH5 | 1003 | CGCAATAGACAAGGACATAACAC | GGTCAAACTGCCCATACTTG |
| CLDN5 | 7122 | TTCGCCAACATTGTCGTCC | TCTTCTTGTCGTAGTCGCCG |
| INSR | 3643 | TGTTCATCCTCTGATTCTCTG | GCTTAGATGTTCCCAAAGTC |
| JAM2 | 58494 | GCTCTAGAATAGACTTCCATGTCCTGCC | GGCAGGACATGGAAGTCTATTCTAGAG |
| OCLN | 100506658 | CTCGAGAAAGTGCTGAGTGCCTGGAC | AAGCTTTCGGTGACCAATTCACCTGA |
| POU5F1 | 5460 | CCTGAAGCAGAAGAGGATCACC | AAAGCGGCAGATGGTCGTTTGG |
| RAGE | 177 | GTAGATTCTGCCTCTGAACTC | CTTCACAGATACTCCCTTCTC |
| RPL30 | 6156 | GCTGGAGTCGATCAACTCTAGG | CCAATTTCGCTTTGCCTTGTC |
| SLC1A1 | 6505 | GTTATTCTAGGTATTGTGCTGG | CTGATGAGATCTAACATGGC |
| SLC1A2 | 6506 | TGCCAACAGAGGACATCAGCCT | CAGCTCAGACTTGGAGAGGTGA |
| SLC1A3 | 6507 | GGTTGCTGCAAGCACTCATCAC | CACGCCATTGTTCTCTTCCAGG |
| TGFB1 | 7040 | AACCCACAACGAAATCTATG | CTTTTAACTTGAGCCTCAGC |
| TJP1 | 7082 | ACCAGTAAGTCGTCCTGATCC | TCGGCCAAATCTTCTCACTCC |
| vWF | 7450 | CCCGAAAGGCCAGGTGTA | AGCAAGCTTCCGGGGACT |

**Supplementary Table 4**

Antibodies used for immunocytochemistry.

- Primary Antibodies

| **Antibody** | **Species** | **Company** | **Catalogue Number** | **Concentration** | **Fixation** |
| --- | --- | --- | --- | --- | --- |
| CD31 | Mouse | Proteintech | 66065-1-Ig | 1:300 | MeOH |
| Claudin-5 | Mouse | Invitrogen | 35-2500 | 1:100 | MeOH |
| Claudin-5 | Rabbit | Abcam | Ab15106 | 1:100 | MeOH |
| Glut-1 | Rabbit | ProteinTech | 21829-1-AP | 1:150 | MeOH |
| VEGF A | Rabbit | Proteintech | 19003-1-AP | 1:500 | MeOH |

- Secondary Antibodies

| **Antibody** | **Species** | **Wavelength (nm)** | **Company** | **Catalogue Number** | **Concentration** |
| --- | --- | --- | --- | --- | --- |
| Anti-mouse | Donkey | 568 | Abcam | A10037 | 1:500 |
| Anti-rabbit | Donkey | 488 | Abcam | A21206 | 1:500 |
